# Molecular characterization of the salivary adenoid cystic carcinoma immune landscape by anatomic subsites

**DOI:** 10.1038/s41598-024-66709-3

**Published:** 2024-07-09

**Authors:** Jason Tasoulas, Travis P. Schrank, Harish Bharambe, Jay Mehta, Steven Johnson, Kimon Divaris, Trevor G. Hackman, Siddharth Sheth, Kedar Kirtane, Juan C. Hernandez-Prera, Christine H. Chung, Wendell G. Yarbrough, Renata Ferrarotto, Natalia Issaeva, Stamatios Theocharis, Antonio L. Amelio

**Affiliations:** 1 Department of Otolaryngology–Head and Neck Surgery, The University of North Carolina at Chapel Hill, Chapel Hill, NC USA; 2 Lineberger Comprehensive Cancer Center, University of North Carolina at Chapel Hill, Chapel Hill, NC USA; 3 Department of Pathology, School of Medicine, National and Kapodistrian University of Athens, Athens, Greece; 4 Department of Tumor Microenvironment and Metastasis, H. Lee Moffitt Cancer Center & Research Institute, 12902 Magnolia Drive, Tampa, FL 33612 USA; 5 Department of Pathology and Laboratory Medicine, School of Medicine, The University of North Carolina at Chapel Hill, Chapel Hill, NC USA; 6 Department of Epidemiology, Gillings School of Global Public Health, The University of North Carolina at Chapel Hill, Chapel Hill, NC USA; 7 Division of Pediatric and Public Health, Adams School of Dentistry, University of North Carolina at Chapel Hill, Chapel Hill, NC USA; 8 Division of Hematology/Oncology, University of North Carolina School of Medicine, Chapel Hill, NC USA; 9 Department of Head and Neck-Endocrine Oncology, H. Lee Moffitt Cancer Center & Research Institute, 12902 Magnolia Drive, Tampa, FL USA; 10 Department of Pathology, H. Lee Moffitt Cancer Center & Research Institute, 12902 Magnolia Drive, Tampa, FL USA; 11 Department of Thoracic/Head and Neck Medical Oncology, The University of Texas MD Anderson Cancer Center, Houston, TX USA

**Keywords:** Adenoid cystic carcinoma (AdCC), Salivary gland cancer (SGC), Tumor biology, Tumor immune microenvironment (TIME), Head and neck cancer, Tumour immunology

## Abstract

Adenoid cystic carcinoma (AdCC) is a slow-growing salivary gland malignancy that relapses frequently. AdCCs of the submandibular gland exhibit unique differences in prognosis and treatment response to adjuvant radiotherapy compared to other sites, yet the role of tumor anatomic subsite on gene expression and tumor immune microenvironment (TIME) composition remains unclear. We used 87 samples, including 48 samples (27 AdCC and 21 normal salivary gland tissue samples) from 4 publicly available AdCC RNA sequencing datasets, a validation set of 33 minor gland AdCCs, and 39 samples from an in-house cohort (30 AdCC and 9 normal salivary gland samples). RNA sequencing data were used for single sample gene set enrichment analysis and TIME deconvolution. Quantitative PCR and multiplex immunofluorescence were performed on the in-house cohort. Wilcoxon rank-sum, nonparametric equality-of-medians tests and linear regression models were used to evaluate tumor subsite differences. AdCCs of different anatomic subsites including parotid, submandibular, sublingual, and minor salivary glands differed with respect to expression of several key tumorigenic pathways. Among the three major salivary glands, the reactive oxygen species (ROS)/nuclear factor erythroid 2-related factor 2 (NRF2) pathway signature was significantly underexpressed in AdCC of submandibular compared to parotid and sublingual glands while this association was not observed among normal glands. Additionally, the NRF2 pathway, whose expression was associated with favorable overall survival, was overexpressed in AdCCs of parotid gland compared to minor and submandibular glands. The TIME deconvolution identified differences in CD4^+^ T cell populations between AdCC of major and minor glands and natural killer (NK) cells among AdCC of minor, submandibular, and parotid glands while plasma cells were enriched in normal submandibular glands compared to other normal gland controls. Our data reveal key molecular differences in AdCC of different anatomic subsites. The ROS and NRF2 pathways are underexpressed in submandibular and minor AdCCs compared to parotid gland AdCCs, and NRF2 pathway expression is associated with favorable overall survival. The CD4^+^ T, NK, and plasma cell populations also vary by tumor subsites, suggesting that the observed submandibular AdCC tumor-intrinsic pathway differences may be responsible for influencing the TIME composition and survival differences.

## Introduction

Adenoid cystic carcinoma (AdCC) is a rare malignancy of the salivary glands, comprising less than 1% of all head and neck cancers^1^. The tumor has an indolent but protracted clinical course and is remarkable for frequent perineural invasion and distant metastases^2^. The standard of care includes *en bloc* resection of the primary cancer with or without adjuvant radiotherapy^3^. In the case of parotid gland cancers, this includes resection of the primary tumor with a cuff of normal parotid tissue and may require sacrificing the facial nerve if there is clinical involvement. In the case of submandibular and minor salivary gland tumors with an increased risk of nodal metastases, a neck dissection is often performed in addition to complete resection of the primary tumors. However, this approach is often hampered by the extent of local invasion which may involve facial structures that are challenging to restore the function and cosmesis. While radiation therapy has known to be effective in the management of salivary gland cancers (SGC) including AdCC, but the role of chemotherapy and targeted therapy is limited^4–6^.

Recent evidence supports an etiologic link between genes of the *myb* family transcription factors *MYB* and *MYBL1* and the transcription factor *NFIB* and AdCC^7–10^. The role of *MYB* and *MYBL1* was initially identified in the context of AdCC specific gene fusions with *NFIB* and less frequently with other genes (i.e., *RAD51* and *TGFBR3*). However, while *MYB* or *MYBL1* are overexpressed in up to 80% of AdCC cases, fusions of these genes are also reported as the key oncogenic driver in these tumors^9,11^. Specifically, fusion detection varies by applied assays, but next generation sequencing studies have identified *MYB* or *MYBL1* rearrangements in > 60% of the examined cases^12^. The non-universal overexpression of *MYB* and *MYBL1* suggests presence of alternative molecular drivers in the tumor formation and progression of AdCC.

Histologically, AdCC consists of two cellular components, epithelial (*TP63*^−^/*NOTCH1*^+^) and myoepithelial (*TP63*^+^/*NOTCH1*^−^), and forms three main growth patterns: cribriform, tubular, and solid^1,11^. Both cell types are present in cribriform and tubular AdCC. However, the solid pattern which is associated with worse prognosis^13^ is often characterized by loss of myoepithelial features and formation of solid sheets and nests of *TP63*^−^/*NOTCH1*^+^ malignant epithelial cells separated by fibrous diaphragms^11^.

Despite the progress in the understanding of AdCC pathobiology, the impact of primary tumor sites on survival and response to treatment is relatively understudied. We and others have previously shown that AdCC is a salivary gland malignancy exhibiting heterogeneity in prognosis and treatment response^14–19^. Also, little is known about the composition of AdCC tumor immune microenvironment (TIME) and how it might be affected by tumor sites^20^. Most of the available information on the AdCC tumor microenvironment emanates from in vitro experiments of cell lines and xenografts^21–27^ with only a few studies analyzing primary tumors^28–33^. While these studies indicated that there might be some inter-tumor heterogeneity in the TIME composition of AdCC, it is unclear whether it represents site-specific differences.

To address this knowledge gap, we performed a comprehensive analysis of AdCC originating from different sites to evaluate differences in survival and TIME composition.

## Materials and methods

### Bulk RNA-seq analysis

Publicly available datasets of bulk RNA sequenced AdCC and normal major and minor salivary glands were used to evaluate site-specific differences in AdCC (Table 1)^30,34–36^. For homogeneity purposes, only studies using Illumina platforms were considered eligible for further processing. Two datasets (n = 38) of AdCCs and 2 datasets of normal, adult major and minor salivary glands were analyzed. In the AdCC cohort, 11 metastatic tumors were excluded, and only primary tumors were included in the analysis. RNA sequencing data were quantified with Salmon, transcript-level abundance estimates were aggregated to per-gene level estimates using the tximport R package^37^. Data were harmonized between datasets/cohorts using identical quantification procedures, filtering and normalization performed simultaneously on the pooled data. Low-expressed genes were filtered out, retaining only genes with ≥ 2 count per million (cpm) reads in half of samples. TMM library size normalization was applied as implemented in the edgeR R-package^38^. The expected effects of data filtering and normalization procedures were confirmed by direct visualization (Supplemental Fig. 1A). The biological groups were recapitulated in the pooled data as evidenced in PCA dimensional reduction plots (Supplemental Fig. 1B). A previously described cohort of minor gland AdCCs analyzed with bulk RNA sequencing was used to further investigate some of the associations identified in the pooled cohort of RNA-sequencing analyzed AdCCs^33^. These reads were processed separately, as previously described, and not pooled with the other datasets.

**Table 1:** Descriptive information of the 4 publicly available RNA sequencing datasets that were included in this study.

Original report	Platform	Type	Sample size	Sample condition	Reads (millions)	Ends	Base pairs
Linxweiler et al.^30^	Illumina	Tumor	20	FF	170–321	Paired	50
Rettig et al.^34^	Illumina	Tumor	18	FF	> 50	Paired	100
Oyelakin et al.^35^	Illumina	Normal (minor)	8	FFPE		Paired	
Saitou et al.^36^	Illumina	Normal (major)	13	FF		Paired	150

FF: Fresh frozen; FFPE: Formalin-fixed, paraffin embedded.

**Figure 1:**
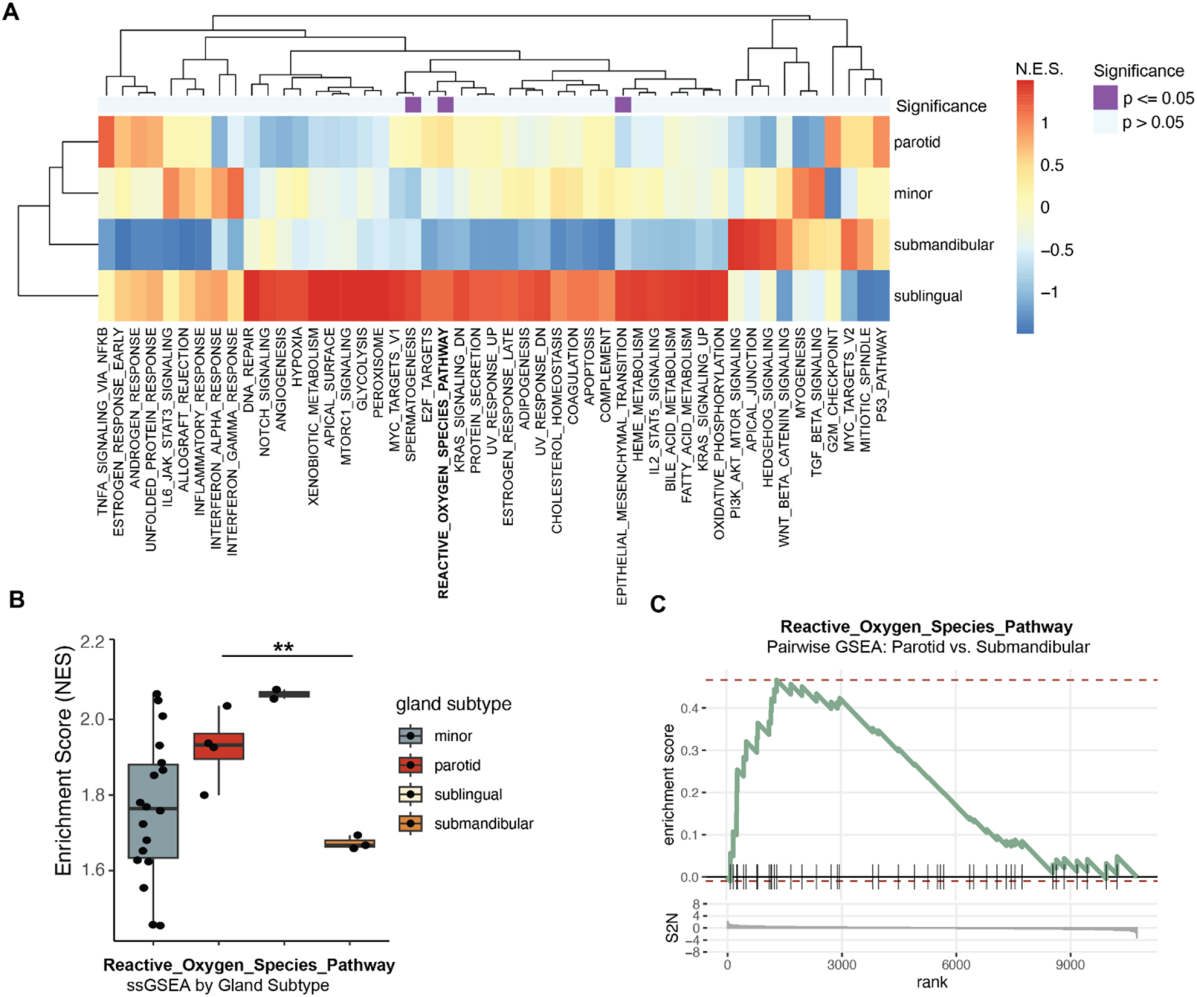
Hallmark gene signature analysis in adenoid cystic carcinoma by gland type (minor vs. major—submandibular, sublingual, parotid). (**A**) Heatmap of gene set enrichment analysis (GSEA) of adenoid cystic carcinomas according to anatomic subsite. (**B**) Boxplot for the normalized enrichment score of the reactive oxygen species (ROS) hallmark signature between adenoid cystic carcinomas according to anatomic subsite. (**C**). GSEA plots of the ROS pathway enrichment score and signal-to-noise (S2N) ratio for parotid gland versus submandibular AdCCs.

### Single sample gene set enrichment analysis and gene set enrichment analysis

Single sample gene set enrichment analysis (ssGSEA) was used to evaluate signatures derived from Human Molecular Signatures Database (MSigDB) Hallmark and in house oncologic signatures^39,40^. ssGSEA was performed based on the above-described pre-processed read counts, which were converted to cpm and log_2_ transformed (logCPM). For ssGSEA analyses, post-processed gene expression, in normalized logCPM were directly used as a rank metrics. For comparative group-based GSEA, genes were ranked according to signal-to-noise ratio as defined by the Broad Institute GSEA software using in house scripts, the R-project fgsea package was used to for GSEA statistics and significance calculation^41^. Test gene sets (Hallmark and Oncogenic gene sets from the MSigDB, Broad Institute) were downloaded from the MSIG data bank via the msigdbr R-project package^42,43^. ssGSEA differences between AdCC subsites were evaluated using Wilcoxon rank-sum and nonparametric equality-of-medians tests using a conventional p < 0.05 statistical significance criterion. In the validation dataset, growth pattern groups with 5 or less samples were excluded from further analysis. AdCCs characterized as tubular (n = 5) and unknown (n = 5) were excluded, and GSEA was performed between cribriform and solid AdCCs. A conventional false discovery rate (FDR) q-value < 0.05 significance criterion was used.

### Tumor immune microenvironment analysis

Tumor immune microenvironment deconvolution of each sample included in the study from all cohorts was performed with the EcoTyper pipeline, which is a non-negative matrix factorization approach trained on single cell RNA sequencing data^44^. The EcoTyper carcinoma pipeline was run in “recovery mode” with default settings on the harmonized per-gene TPM estimates, described above. Cell types were quantified as the sum of their substate intensities. Cell types were quantified as a log fraction, log_2_((microenvironment cell)/(epithelial cell)). TIME composition differences between AdCC subsites were tested using Wilcoxon rank-sum and nonparametric equality-of-medians tests using a conventional p < 0.05 statistical significance criterion.

### Clinical samples

A retrospective in-house cohort of 39 participants, consisting of 30 AdCC patients (10 minor, 10 submandibular, 10 parotid gland AdCC) and 9 healthy normal controls (3 minor, 3 submandibular, 3 parotid) with archival tissue was identified. All participants had fresh-frozen paraffin embedded tissue blocks available, dated from 2016 to 2022. AdCC patients had a diagnosis of primary, non-metastatic AdCC treated surgically, with or without adjuvant radiotherapy. All tissue blocks were obtained from primary surgical resections. Pathology slides from all cases, (hematoxylin and eosin; H&E) were reviewed by a head and neck pathologist who confirmed the diagnoses. Normal tissues were obtained from negative excision biopsies of patients evaluated for benign conditions. All controls had no underlying immune-related disorders. H&E slides were reviewed by a head and neck pathologist who characterized them as healthy salivary gland tissue. The study was approved by the Institutional Review Board of the University of North Carolina at Chapel Hill (IRB: 23-0245).

The AdCC and normal tissue control cohorts were fully clinically annotated (Table 2). Characteristics of interest included age at diagnosis, sex, primary site (parotid, submandibular, minor), growth pattern (cribriform and/or tubular, solid), presence of solid component (defined as ≥ 30% solid growth pattern), T-stage (1–4), N-stage (0–3), perineural invasion (no, yes), lymphovascular invasion (no, yes), margin status (negative, positive), relapse status (no, yes), status (alive, dead) and overall survival (days).

**Table 2:** Demographic and clinicopathologic information of the in-house cohort of adenoid cystic carcinoma patients.

Tumor site	Parotid	Submand	Minor	Total	col. %	p-value
Age, years (SD)	59 (17)	52 (16)	61 (12)	57 (15)		
Sex						0.287
Female	5	8	5	18	80	
Male	5	2	5	12	20	
Histological type						0.271
Cribriform/tubular	6	9	8	7	80	
Solid	4	1	2	23	20	
T-stage						0.056
T1	1	3	2	6	20	
T2	2	3	0	5	17	
T3	2	4	1	7	23	
T4	5	0	7	12	40	
N-stage						0.315
N0	8	10	8	26	87	
N1	0	0	0	0	0	
N2	2	0	2	4	13	
N3	0	0	0	0	0	
Perineural invasion						0.621
No	4	3	2	9	30	
Yes	6	7	8	21	70	
Lymphovascular invasion						0.271
No	9	8	6	23	77	
Yes	1	2	4	7	23	
Margins						0.866
Negative	4	4	3	11	37	
Positive	6	6	7	19	63	

### Tissue sectioning, RNA isolation, and real-time qPCR

H&E slides from all available blocks were reviewed by a head and neck pathologist (SJ) to select tissue blocks with maximum enrichment in tumor cells. Sectioning was performed at the UNC Lineberger Comprehensive Cancer Center, Pathology Services Core. Four 3μm sections per block were used for H&E and multiplex immunofluorescence. Five 10μm sections per block were used for RNA extraction. RNA extraction was performed at UNC Lineberger Comprehensive Cancer Center, Translational Genomics Lab using Thermo Scientific KingFisher Flex (Cat 5400610) and Applied Biosystems MagMAX FFPE DNA/RNA Ultra Kit (Cat A31881). RNA quality control was performed using Agilent TapeStation 4200 (Cat G2991AA), Agilent RNA ScreenTape Analysis (Cat 5067-5576) (RNA quality). RNA was quantified using the Qubit RNA HS kit (Thermo Fischer Scientific, Wilmington, DE, USA). cDNA was synthesized using the Superscript IV Vilo cDNA Synthesis Kit (#AB1453B, Thermo Fischer Scientific, Wilmington, DE) according to the manufacturer’s instructions. cDNA was quantified using the Qubit ssDNA (Thermo Fischer Scientific, Wilmington, DE, USA).

The expression of *NRF2* and its targets *NQO1*, *TXNRD1* and *HMOX1* was analyzed by SYBR Green method on an Applied Biosystems QuantStudio 5 Real-Time PCR system (Thermo Fischer Scientific, Wilmington, DE) using 10–20 ng of cDNA per reaction. RT-qPCR reactions were done in triplicates using PowerUp SYBR™ Green PCR Master Mix (#4367659, Thermo Fischer Scientific, Wilmington, DE). The Relative Quantity (RQ) was estimated as RQ = 2^−(Ct^_test_^–Ct^_control_^)^ X 100 using RPL23 expression as an endogenous control. Samples with RPL3 Ct values < 28 were considered for analysis. Fold changes in the expression of *NRF2* and its target genes were calculated with respect to the expression of respective genes in matched normal tissues.

### Multiplex immunofluorescence stains

Multiplex immunofluorescence (mIF) was performed at the UNC Pathology Services Core (Supplemental Fig. 2). Tissue sections were prepared for both H&E and IF staining. The H&E slides were scanned, and the images were reviewed by the study pathologist. The pathologist then marked regions of tumor using the drawing tools in WebViewer or ImageScope. These annotations (as XML data) were then copied to subsequent images of tissue sections from the same blocks that were stained for multiplex IF detection. A subset of AdCC cases (n = 9), 3 minor, 3 submandibular and 3 parotid gland AdCCs were selected for analysis for triplex mIF staining as follows.

**Figure 2:**
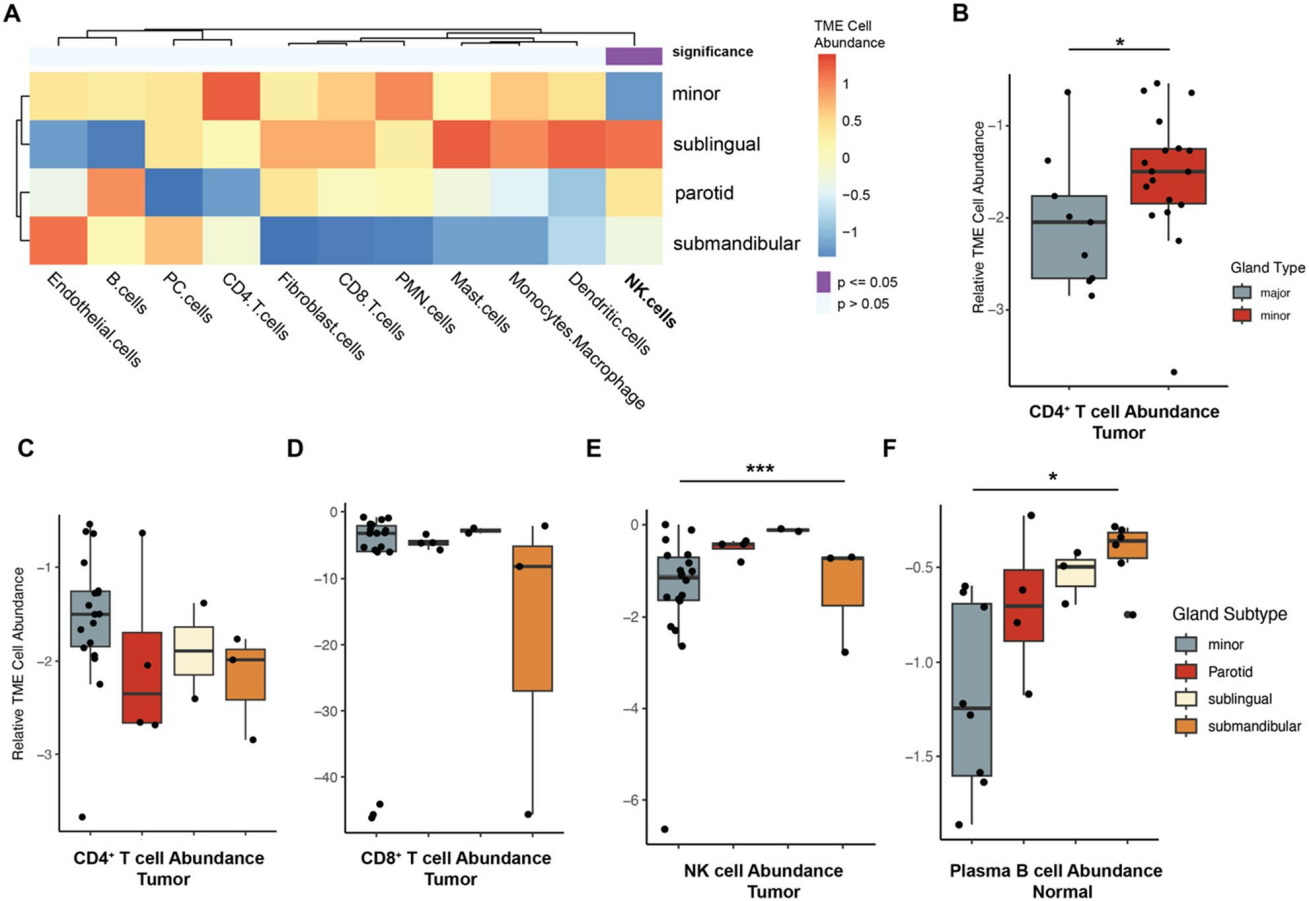
Anatomic subsite-dependent differences in the tumor immune microenvironment. (**A**) Heatmap of the tumor immune microenvironment cell population composition using ECOTYPER software. Boxplots for normalized enrichment of (**B**) CD4^+^ T cells in major versus minor gland adenoid cystic cell carcinomas and (**C**) parotid, submandibular, sublingual and minor gland adenoid cystic cell carcinomas, (**D**) CD8^+^ T cells in parotid, submandibular, sublingual and minor gland adenoid cystic cell carcinomas, (**E**) NK cells between parotid, submandibular, sublingual and minor gland adenoid cystic cell carcinomas, and (**F**) plasma B cells between normal parotid, submandibular, sublingual and minor gland samples. p-values depicted were obtained from Wilcoxon rank-sum and nonparametric equality-of-medians tests.

#### NRF2, CD4^+^ T cell, and CD138^+^ plasma cell stain

Triple IF was performed on paraffin-embedded tissues that were sectioned onto positively charged slides. Tissue sections were labeled for antigens using a triple stain assay. The multiplex combination was with NRF2 (Abcam, ab137550), CD4 (Cell Marque, 104R-25), and CD138 (Leica, PA0088). This IF assay was carried out on the Leica Bond Rx fully automated slide staining system (Leica Biosystems) using the Bond Research Detection kit (DS9455). Slides were deparaffinized in Leica Bond Dewax solution (AR9222), hydrated in Bond Wash solution (AR9590) and sequentially stained for a triplex stain. Briefly, antigen retrieval was accomplished using Bond-epitope retrieval solution 1 pH 6.0 (AR9961). After pretreatment, tissues were blocked, and primary antibodies were diluted as follows: NRF2 at 1:1000, CD4 at 1:200, and CD138 was Ready-to-Use (RTU). RTU secondary antibodies Novolink Post Primary and/or Novolink Polymer (Leica Biosystems, RE7260-CE) were used followed by either TSA Cy5 (Akoya Biosciences, SAT705A001EA), TSA Cy3 (Akoya Biosciences, SAT704A001EA) or Alexa Fluor™ 488 Tyramide Reagent (Thermo Fisher Scientific, B40953) to visualize the target of interest. Nuclei were stained with Hoechst 33258 (Invitrogen). The stained slides were mounted with ProLong Gold antifade reagent (Thermo Fisher Scientific, P36930). Positive and negative controls (no primary antibody) were included in this run.

#### NRF2, CD3^−^CD56^+^ NK cell stain

Triple immunofluorescence (IF) was performed on paraffin-embedded tissues that were sectioned onto positively charged slides. Tissue sections were labeled for antigens using a triple stain assay. The multiplex combination was with NRF2 (Abcam, ab137550), CD56 (Leica, PA0191-U), and CD3 (Leica, NCL-L-CD3-565). This IF assay was carried out on the Leica Bond Rx fully automated slide staining system (Leica Biosystems) using the Bond Research Detection kit (DS9455). Slides were deparaffinized in Leica Bond Dewax solution (AR9222), hydrated in Bond Wash solution (AR9590) and sequentially stained for a triplex stain. Briefly, antigen retrieval was accomplished using Bond-epitope retrieval solution 1 pH 6.0 (AR9961) or Bond-Epitope Retrieval solution 2 pH-9.0 (AR9640). After pretreatment, tissues were blocked, and primary antibodies were diluted as follows: NRF2 at 1:1000, CD56 was Ready-to-Use (RTU), and CD3 at 1:1000. RTU secondary antibodies Novolink Post Primary and/or Novolink Polymer (Leica Biosystems, RE7260-CE) were used followed by either TSA Cy5 (Akoya Biosciences, SAT705A001EA), TSA Cy3 (Akoya Biosciences, SAT704A001EA) or Alexa Fluor™ 488 Tyramide Reagent (Thermo Fisher Scientific, B40953) to visualize the target of interest. Nuclei were stained with Hoechst 33258 (Invitrogen). The stained slides were mounted with ProLong Gold antifade reagent (Thermo Fisher Scientific, P36930). Positive and negative controls (no primary antibody) were included in this run.

All slides were digitized using the Aperio ScanScope FL (Aperio Technologies Inc). The digital images were captured in each channel by 20× objective (0.468 μm/pixel resolution) using line-scan camera technology (U.S. Patent 6,711,283). The adjacent 1 mm stripes captured across the entire slide were aligned into a contiguous digital image by an image composer. Images were archived in PSC’s eSlide Manger database (Leica Biosystems). Digital slides (images) from the ScanScope FL were imported as separate SVS files for each channel along with the Aperio Fused Image (AFI) script. Within the analysis application, it is possible to view all the fluorescent channels merged or individually. The previously drawn annotations were also imported as the tumor regions of interest (ROIs). Once the tumor regions were defined, the algorithm created a margin around each tumor ROI (Supplemental Fig. 3). This margin was 50-um wide with half the width inside the tumor and the other half outside the tumor. With the tumor cores and margins defined, the algorithm proceeded with cellular detection and scoring. This began by selecting up to 12 sample areas from the images. Within each sample area, the nuclei were defined based on the Hoechst stain signal. Each cell was simulated by growing outward from the nucleus by approximately 10-µm. After the cells were segmented, each marker was detected separately. Scoring markers as positive or negative was based on intensity levels. The cutoff value was determined by comparison with control samples and evaluation of background and auto-fluorescence. The data output included number, percentage, and density of all cell populations with or without marker co-expression.

**Figure 3:**
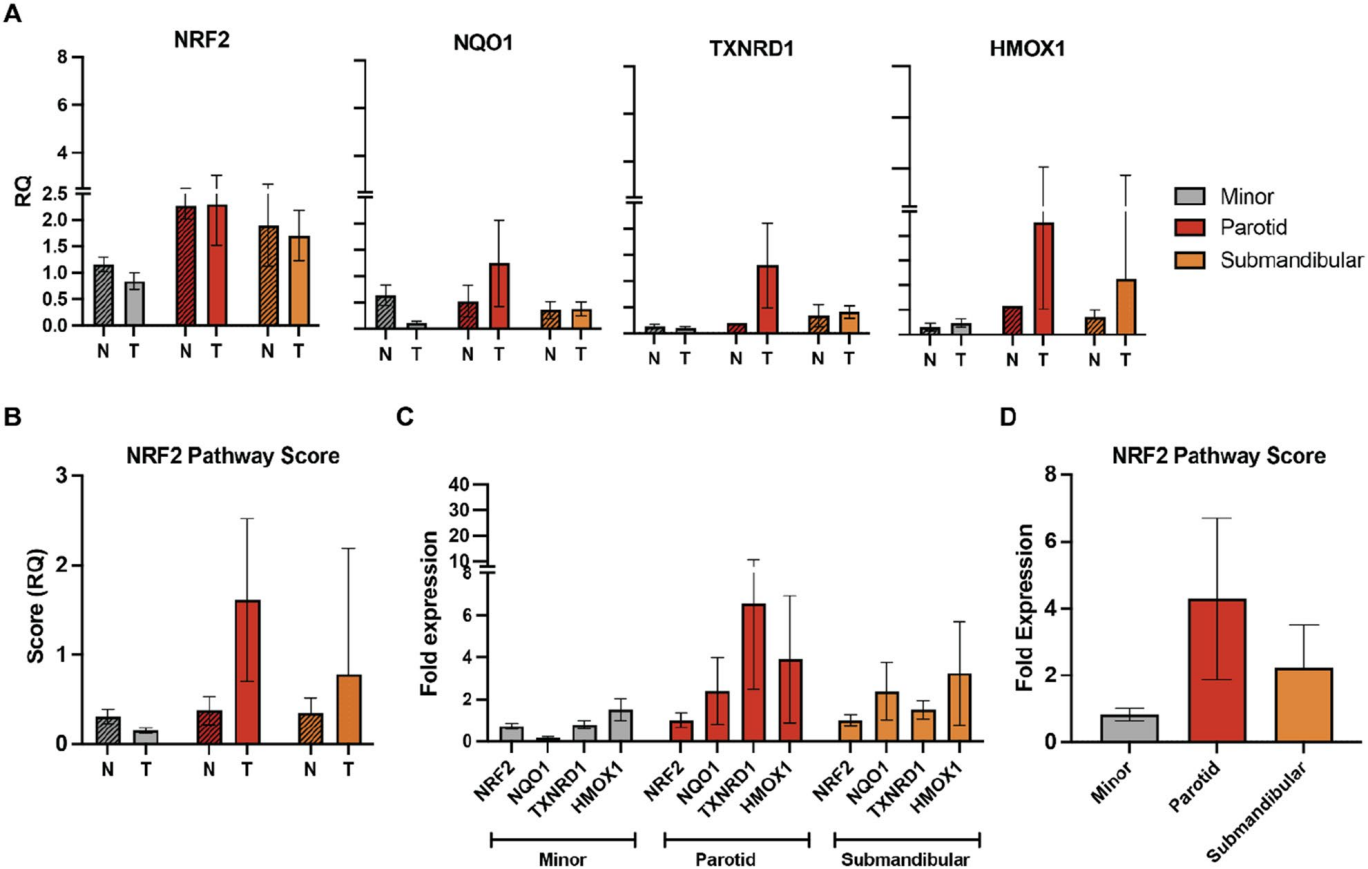
Activation of the NRF2 pathway in parotid gland AdCC. (**A**) Real-time quantitative PCR (qPCR) analysis of canonical NRF2 target gene expression in normal (N) versus tumor (T) samples according to gland subsite. (**B**) NRF2 pathway score analysis (average of *NQO1, TXNRD1* and *HMOX1* expression) according to gland subsite. (**C**) Relative fold expression of *NRF2* and its direct target genes, or (**D**) NRF2 pathway score within AdCC tumors according to gland subsite. Normalization was performed using endogenous *RPL23* expression (Relative Quantification; RQ) and expression of the respective gene in the same site of normal salivary glands (fold expression).

### Statistical analysis

Summary statistics for demographic characteristics were compared using frequencies and means. A non-parametric, Wilcoxon rank sum test was used to compare the levels of enrichment in the ssGSEA and the levels of TIME populations in the EcoTyper analysis. The distribution of the fold change of the NRF2 pathway gene expression was non-normal and thus, the same non-parametric test was used to evaluate differences between salivary gland sites. A *NRF2* pathway score was created estimating the average of three *NRF2* targets: *NQO1*, *TXNRD1*, *HMOX1.* A linear regression model was used to estimate associations between the relative quantitative and fold change expression of NRF2 pathway genes and overall survival. Analyses were carried out using Stata 16.1 (StataCorp LP, College Station, TX, USA) and R (v3.2-7) packages ggplot2^45^, wesanderson^46^, RColorBrewer^47^, and pheatmap^48^.

## Results

### The ROS pathway defines anatomic subsite differences in adenoid cystic carcinoma

We recently discovered that reported differences in AdCC patient survival may be linked to molecular and immunological differences associated with distinct anatomic subsites^14–19^. To investigate the molecular mechanisms that govern these disparate prognoses, we set out to examine the molecular landscape of tumors derived from both minor and major salivary glands using whole-transcriptome data. Specifically, we leveraged RNA sequencing datasets from previously published AdCC studies and separately assembled an in-house, fully clinically annotated AdCC cohort to validate the results of those bioinformatics analyses and elucidate the role that tumor subsite plays on the underlying cancer-associated pathway expression and the TIME composition.

Data from all four eligible RNA sequencing datasets were processed and harmonized (Supplemental Fig. 1A), and the subsequent PCA confirmed overlap in gene expression between AdCC samples compared to normal gland tissues (Supplemental Fig. 1B). After excluding metastatic AdCC samples, we identified different levels of enrichment for several key tumorigenic pathways between AdCCs arising within different anatomic subsites. Gene set enrichment analyses (GSEA) revealed significant differences in several pathways between anatomic sites with the reactive oxygen species (ROS) pathway being of considerable interest (Fig. 1A). ROS signaling is significantly (p < 0.007) repressed in submandibular versus parotid gland AdCCs (Fig. 1B,C). Notably, minor gland AdCCs showed a bi-modal distribution, with a subset of tumors being significantly enriched in the ROS signature (NES > 1.8). These pathway signature differences were not seen in control tissue comparisons, indicating that these features are AdCC-specific.

### ROS pathway enrichment correlates with unique subsite-dependent tumor immune microenvironment profiles and corresponding NRF2 signaling activity

Accumulating evidence supports a role of ROS signaling in regulating immune infiltrates within the TIME^49,50^. Thus, we next performed TIME deconvolution analyses to test whether the differences in ROS pathway observed in distinct AdCC subsites are associated with differences in immune cell composition. EcoTyper was employed as previously described^44^ to identify cell states and cellular communities from the bulk RNA-seq data (Fig. 2A). When comparing minor gland versus major gland tumors, there was a significant (p = 0.041) enrichment of CD4^+^ T cells in minor gland AdCCs (Fig. 2B). When further stratifying major gland tumors by anatomic subsite, however, enrichment of CD4^+^ T cells and CD8^+^ T cells were not significantly different (p = 0.185; p = 0.511) (Fig. 2C,D). We identified a significant (p < 0.005) enrichment of natural killer (NK) cells within parotid and sublingual gland AdCCs compared to submandibular gland tumors (Fig. 2E). Interestingly, comparison to normal control tissues revealed that normal submandibular glands are characterized by a significant (p = 0.045) enrichment of plasma B cells compared to other anatomic subsites (Fig. 2F).

Oxidative stress induced by activation of ROS signaling is known to be regulated by the nuclear factor erythroid 2-related factor 2 (*NRF2*) pathway^51^. *NRF2* is a transcription factor that governs the gene expression of endogenous antioxidant synthesis and *ROS*-eliminating enzymes, and therefore is a master regulator of neutralizing cellular *ROS* and restoring redox balance. Notably, ROS-associated changes within the TIME can induce metabolic reprogramming and pro-inflammatory cytokine production in several immune cells, including T cell subsets^52^. To test if the NRF2 pathway is differentially regulated in AdCCs according to anatomic subsite, we next performed a comprehensive real-time qPCR analysis of NRF2 target gene expression using our in-house cohort of parotid, submandibular, and minor gland AdCCs (Fig. 3). While NRF2 expression levels were similar between normal salivary gland tissues and AdCC tumors from each anatomic subsite, analysis of the canonical targets *NQO1, TXNRD1,* and *HMOX1* which are directly regulated by NRF2 revealed that parotid gland AdCCs have elevated levels of the expression (Fig. 3A). Specifically, parotid AdCC had a higher mean NRF2 pathway score RQ (1.61), and a higher mean NRF2 pathway score fold (4.29), compared to AdCC of submandibular gland (0.78; 2.22) and minor gland (0.15; 0.83). An NRF2 pathway score (average of *NQO1, TXNRD1* and *HMOX1* expression) further highlighted the elevated NRF2 signaling levels in AdCC of parotid glands (Fig. 3B). Comparison of AdCC across each subsite further supports these findings and demonstrates that AdCC of parotid glands can have 2–sixfold higher expression of these NRF2 targets compared to minor and submandibular glands (Fig. 3C,D).

### NRF2 expression is associated with NK cell infiltration and improved survival of patients with AdCC of parotid glands

**Figure 4:**
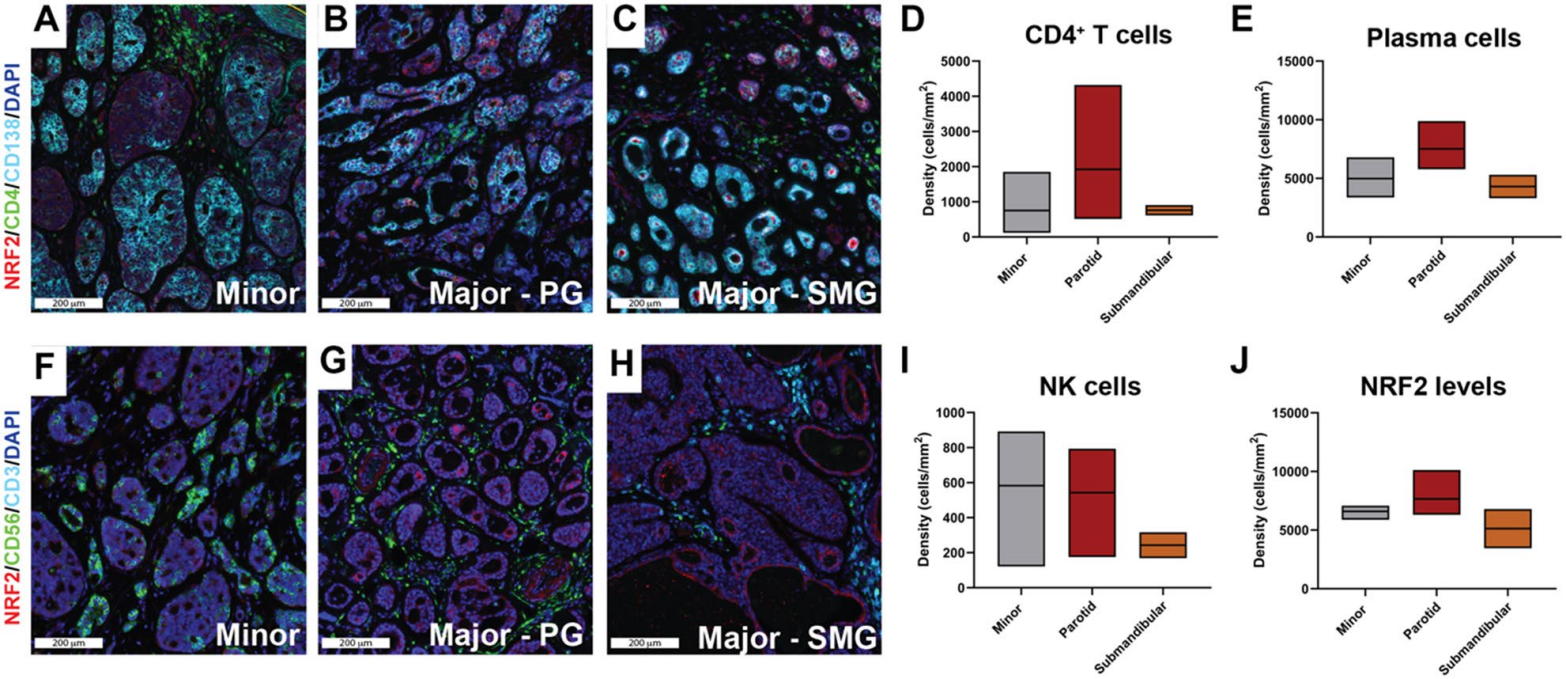
ROS/NRF2 levels associate with unique TIME landscapes across AdCC anatomic subsites. (**A**–**C**) Multiplex IF staining for NRF2 (red), CD4+ T cells (green), and CD138+ Plasma B cells (cyan) in minor (**A**), parotid (**B**) and submandibular gland (**C**) AdCCs. Floating bars plots of CD4^+^ T-cell (**D**), and plasma cell density (cells/mm^2^) with means (**E**), in minor, parotid and submandibular gland adenoid cystic carcinomas (AdCCs). (**F**–**H**) Multiplex IF staining for NRF2 (red) and CD3−/CD56+ Natural Killer (NK) cells (green) in minor (**F**), parotid (**G**) and submandibular gland (**H**) AdCCs. Scale bar = 200 nm. Floating bars plots of NK cell (**I**) and NRF2^+^ cell density (cells/mm^2^) (**J**) with means, in minor, parotid and submandibular gland AdCCs. PG: parotid gland; SMG: submandibular gland.

To further investigate associations between the ROS/NRF2 pathway with composition and spatial distribution of immune cell populations according to AdCC subsites, we also performed mIF on a subset of our in-house cohort samples (3 parotid, 3 submandibular, and 3 minor gland AdCCs). This mIF validated the immune cell types identified in the computational analysis of the TIME (Fig. 2) and indicated that AdCC of minor glands are characterized by infiltration of CD4^+^ T cells compared to submandibular glands although this trend was not statistically significant (Fig. 4A–D). Interestingly, parotid gland AdCCs in this cohort also displayed elevated CD4^+^ T cell infiltration which appears to support one of the apparent outlier samples of the EcoTyper analysis (Fig. 2C). In contrast to EcoTyper results, significant staining of CD138^+^ is observed across all tumors (Fig. 4A–C). However, while this is a well-established Plasma B cell marker^53^, this marker is also known to be expressed on tumor epithelial cells and may explain the observed staining pattern within the tumor islands of our cohort^53^. Furthermore, mIF also included analysis of CD3^−^/CD56^+^ NK cell infiltrates and NRF2 expression. CD3^−^/CD56^+^ NK cells are primarily observed in AdCC of parotid glands compared to other gland subsites (Fig. 4D–F). Analysis of NRF2 revealed significant staining levels in AdCC of parotid glands compared to other gland subsites supporting the elevated ROS/NRF2 pathway observed at this subsite (Figs. 3D and 4J).

Collectively, these findings suggest that the elevated levels of CD4^+^ T, CD138^+^ Plasma B cells, and CD56^+^ NK cells in parotid gland AdCCs are associated with a high ROS signature and elevated NRF2 target gene expression that may be linked to the favorable outcomes observed in patients with parotid gland versus submandibular gland AdCCs. Thus, we next examined the association between overall survival and the NRF2 pathway score, using a univariable linear regression model. Both NRF2 pathway score RQ (coefficient: 13.9, p = 0.042), and a higher mean NRF2 pathway score fold (coefficient 5.53, p = 0.028) were significantly associated with overall survival supporting the notion that the unique molecular and TIME differences characteristic of parotid (↑ ROS/NRF2, ↑ NK cells) versus submandibular (↓ ROS/NRF2, ↓ NK cells) gland AdCCs is in part responsible for the disparate prognoses associated with these two anatomic subsites.

### Favorable minor gland AdCC growth patterns display ROS pathway enrichment

**Figure 5:**
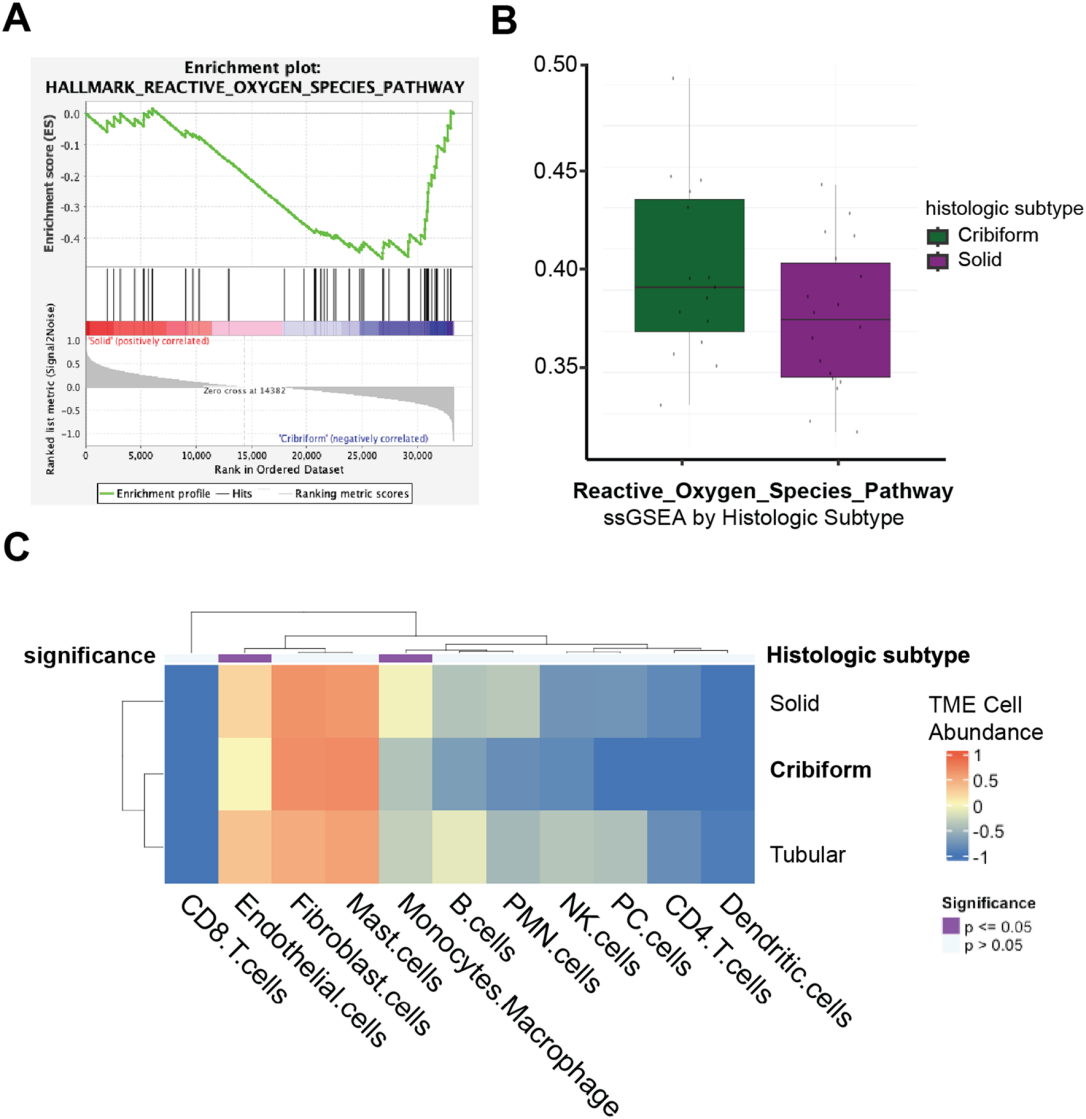
Growth pattern-dependent differences in minor gland AdCCs. (**A)** GSEA and (**B**) ssGSEA plots of the ROS pathway enrichment score and signal-to-noise (S2N) ratio for cribriform versus solid minor gland AdCCs. (**C**) Heatmap of the tumor immune microenvironment cell population composition using ECOTYPER software, by growth pattern.

To further investigate the apparent bi-modal distribution in ROS signature (Fig. 1B) and CD56^+^ NK cell (Fig. 4I) enrichment of minor gland AdCCs, we used a previously described validation dataset of bulk RNA-sequenced minor gland AdCCs^33^. Specifically, processed reads of minor gland AdCC subsites were used, and non-minor gland AdCC samples (i.e., major gland AdCCs) were excluded from analysis. GSEA identified different levels of ROS pathway enrichment between minor gland growth patterns, with the cribriform subtype displaying significant ROS pathway activation [GSEA False Discovery Rate (FDR) q-value = 0.003, nominal p-value = 0.014] relative to the solid subtype (Fig. 5A). Solid subtype has been associated with poor prognosis^13^. The negative association between solid subtype AdCC and NRF2/ROS further strengthens the positive association between NRF2/ROS and overall survival, that is likely underlying the survival differences between different anatomic sites. Notably, the ssGSEA showed a similar trend, though not significant (p = 0.145), between cribriform and solid minor gland AdCCs (Fig. 5B).

We next performed TIME deconvolution analyses to test whether the differences in ROS pathway enrichment observed in cribriform and solid AdCC growth patterns are associated with differences in immune cell composition. EcoTyper was used and the bulk RNA sequencing validation dataset was used to identify tumor immune cell types (Fig. 5C). When comparing across minor gland growth patterns, the enrichment of endothelial cells (p = 0.006) and monocytes and macrophages (p = 0.001) was significantly different. These data suggest that ROS pathway activation within minor gland subsites influences different immune cell subsets compared to those observed at major gland subsites.

## Discussion

The systemic options for the management of AdCC are limited. No targeted or immune-checkpoint inhibitor therapy has proven to be clinically effective, but there is an active investigation towards multiple different directions^6^. Adenoid cystic carcinoma is known to be one of the most immunologically “cold” salivary gland malignancies^54^, and this potentially explains the minimal response to immune checkpoint inhibition approaches in the recurrent and metastatic setting. Most of the trials pool together salivary gland malignancies of different histology and primary sites, and the response to immune checkpoint inhibitors has been relatively poor. To date, 1/325 complete response (0.3%) and 17/325 partial responses (5%) have been recorded among 9 prospective trials that included patients diagnosed with AdCC^55–63^. Thus, new approaches aiming to improve our understanding of the complex interplay between AdCC and its TIME and potentially guide novel treatment approaches and disease management are necessary in order to improve clinical outcomes.

In this study we provide evidence that previously observed subsite-specific survival differences are associated with unique molecular and immunological landscapes. Specifically, we identify site-specific NRF2 pathway expression differences among parotid, submandibular, and minor gland AdCCs, that are associated with overall survival. Specifically, we found that submandibular gland and minor gland AdCCs have lower NRF2 pathway gene expression compared to parotid gland AdCCs. Further, we report that NRF2 pathway gene expression is positively associated with overall survival, suggesting a link between activation of this pathway and site-specific survival differences observed in AdCC. Also, we identify TIME differences between the different AdCC subsites using bulk RNA sequencing TIME deconvolution methods and evaluate the spatial distribution of the identified immune cell populations using mIF. A recent report demonstrated that maxillary sinus and palate minor gland AdCCs were associated with a unique molecular subtype and worse prognosis compared to parotid AdCCs^10^. In this direction, this study further defines the role of tumor anatomic subsite as a parameter affecting the molecular background and the TIME of AdCC.

Anatomic location is known to be associated with survival in several malignancies including head and neck^64^, and non-head and neck tumors like melanoma, colorectal carcinoma, and pancreatic carcinoma^65–67^. Besides informing prognosis, anatomic location appears to be associated with distinct genetic alterations and subsequent pathway activation. In melanoma, different anatomic sites were associated with different oncogenic drivers and expression profiles. Specifically, cutaneous melanomas and acral lentiginous melanoma cells appeared to be driven by *BRAF* mutations and *CRKL* amplifications inducing the HOX13-IGF signaling pathway, respectively ^68^. In AdCC, the role of tumor site on survival has been reported by multiple studies^14–19^. Specifically, submandibular gland and minor gland have been identified as locations independently associated with poor prognosis and increased risk for distant metastasis^14–16,18,69^. Moreover, future studies are needed to address whether the differences in subsite-specific survival are specific to AdCC or if this is more generally observed with other salivary gland carcinomas.

There is accumulating evidence in support of the role for ROS in carcinogenesis and the TIME^49,50^. ROS appears to affect both cancer and stromal cells by inducing metabolic reprograming in a dose dependent fashion. Several studies have shown that increased ROS production in cancer cells can promote activation of immune responses^50,70^. Moreover, the extent of ROS activation within immune cells has also been shown to play a crucial role in their function^50^. Moderate increases in ROS levels result to mild oxidative stress and thus induce oncogenic metabolism, tumorigenesis, angiogenesis and metastasis, but high ROS levels result to high oxidative stress and cytotoxic cell metabolism, inducing apoptosis, ferroptosis, autophagy and necrosis as well as immune deregulation^50^. Thus, a critical balance of ROS activity within both tumor cells and stromal cells is involved in regulating immune function within the TIME.

NRF2 is regulated by ROS pathway activation and these two gene expression signatures overlap significantly. While the NRF2 pathway has been associated with various hallmarks of cancer, its activity is closely associated with ROS levels^51^. NRF2 signaling in cancer cells has been shown to recruit NK cells to the TME through direct regulation of IL17D^71,72^. In our study, we observed higher infiltration of CD3−/CD56+ NK cells in the Parotid gland AdCCs which have high NRF2 pathway scores. Higher levels of ROS in tumors can dampen NK cell function. Similarly, pharmacologic activation of NRF2 has been previously shown to maintain NK cell anti-tumor response^73,74^. Therefore, optimal regulation of ROS by NRF2 can preserve NK cell function and potentially enhance overall survival. While there is limited evidence on the role of ROS in AdCC, elevated ROS levels has been associated with reduced clonogenic survival and increased autophagy in AdCC cells^75^. Our results further support this finding and suggest that differences in ROS levels are associated with different tumor subsites, which may directly influence patient survival. Further, NRF2 pathway activation that also appeared to be higher in parotid AdCCs was associated with improved survival. This activation could be a result of the increased ROS levels in these tumors, explaining the improved survival of these patients. Also, ROS pathway was enriched in cribriform compared to solid minor gland AdCCs, further strengthening the proposed association between ROS/NRF2 signaling and improved survival.

Limited evidence indicates that TIME differences exist between AdCC tumor sites. For example, submandibular gland AdCCs appear to have higher vascular density, which could indicate a potential pathway for dissemination and result in higher rates of distant metastases^18^. Our study provides valuable insights into molecular and cellular mechanisms that could be associated with differences in the biological behavior of AdCCs across different anatomic subsites. ROS and NRF2 pathways are known regulators of TIME composition and activity^49–51^, and variation in their levels may explain the site-specific differences in certain patient populations. These findings have the potential to improve management strategies for this disease. As of now, systemic treatments have not achieved meaningful improvement of survival, and there is no FDA approved agent for treating AdCC^76^. However, multiple targeted agents and immunotherapy combinations are currently actively evaluated in AdCC^77^. Tumor location is emerging as a factor that could potentially affect treatment response through its associated tumor-intrinsic and tumor-microenvironment related differences. Future studies evaluating compounds targeting AdCC cells should consider the site of origin, and the potentially unique features of neoplasms derived from different glands.

Despite its novelty and merits, the present study has some limitations. First, AdCC is a rare tumor and thus, the assembly of large cohorts is not feasible. While the size of cohorts used in the present study (in-house and external) is comparable to most of the other published AdCC cohorts, the overall number of cases are relatively small. When stratifying by tumor site, the number of cases per group decreases further, reducing statistical power, and hampering opportunities for illuminating between-group differences. Further, while the role of tumor location is becoming quite clear through the present study, the location of minor glands was not accounted for in any of the datasets used, and all minor gland AdCCs were pooled together in one category. Finally, the small number of study participants in each RNA-seq dataset and the lack of normal salivary gland controls in the AdCC RNA-seq datasets required us to pool these different datasets. This choice poses profound challenges in data harmonization. Despite our best efforts to limit them, batch effects cannot be entirely removed, and thus all analyses resulting from the pooled RNA-seq dataset should be interpreted with caution. Future studies, investigating a single, clinically annotated cohort of primary AdCCs from different sites and normal gland controls, that are powered to detect differences between tumor sites, will be able to increase our understanding on the role of tumor site in AdCC. Besides the potential bias from batch effects, the present study did not include sublingual AdCCs in most analyses and thus was unable to investigate whether they also exhibit unique molecular and cellular features. Also, epithelial-mesenchymal transition (EMT) was identified to be differentially expressed between the various anatomic sites included in this study. While EMT is known to be a key process in carcinogenesis^78^ and an important prognostic factor in various other malignancies^79^, these associations were not evaluated in this study.

In conclusion, our study offers evidence of site-specific cellular and molecular features of AdCC, which are also associated with survival. ROS and NRF2 pathways are underexpressed in AdCC of submandibular and minor glands compared to parotid glands, and NRF2 pathway expression is associated with favorable overall survival. Also, TIME composition varied by tumor site with AdCC of the minor glands having more CD4^+^ T cells than major glands, the submandibular glands being more enriched for plasma cells, and the parotid gland being more enriched for NK cells. Given the known role of ROS and NRF2 pathways in TIME composition and its interplay with cancer cells, TIME composition could be associated with the observed differences in the expression of these pathways.

### Supplementary Information

Supplementary Information.

## Data Availability

The datasets generated and/or analyzed during the current study are available in the NCBI Sequence Read Archive (SRA) repository, the European Genome-Phenome Archive (EGA), the NCBI Gene Expression Omnibus (GEO) repository, or Mendeley Data under accession numbers: https://www.ncbi.nlm.nih.gov/sra/docs/, https://ega-archive.org/studies/EGAS00001002812, https://www.ncbi.nlm.nih.gov/gds, https://data.mendeley.com/datasets/6sbv7bpj5n/1. SRP109264, SRP096726, and PRJNA601423—Linxweiler et al. ^30^, EGAS00001003959 and EGAS00001008192—Rettig et al. ^34^, GSE157159—Oyelakin et al. ^35^, GSE143702—Saitou et al. ^36^, 10.17632/6sbv7bpj5n.1 et al.—Ferrarotto ^33^.
