# Supplementary material for: Molecular characterization of the salivary adenoid cystic carcinoma immune landscape by anatomic subsites
Source: Sci Rep. 2024 Jul 9;14:15821. doi: 10.1038/s41598-024-66709-3 (PMC11233590; doi:10.1038/s41598-024-66709-3)
Supplement: Supplementary file 1 — Supplementary Information. [file 41598_2024_66709_MOESM1_ESM.docx]

**Molecular Characterization of the Salivary Adenoid Cystic Carcinoma Tumor Immune Landscape by Anatomic Subsite**

Jason Tasoulas MD, DMD^1,2,3^, Travis P. Schrank MD, PhD^1,2^, Harish Bharambe PhD^4^, Jay Mehta MSc^4^, Steven Johnson MD^5^, Kimon Divaris DDS, PhD^6, 7^, Trevor G. Hackman MD^1^, Siddharth Sheth DO, MPH^8^, Kedar Kirtane MD^9^, Juan C. Hernandez-Prera MD^10^, Christine H. Chung MD^9^, Wendell G Yarbrough MD, MMHC^1,2,5^, Renata Ferrarotto MD^11^, Natalia Issaeva PhD^1,2^ ,Stamatios Theocharis MD, PhD^3^, Antonio L. Amelio PhD^1,4, 9*^

^1^Department of Otolaryngology – Head and Neck Surgery, The University of North Carolina at Chapel Hill, Chapel Hill, NC, USA

^2^Lineberger Comprehensive Cancer Center, University of North Carolina at Chapel Hill, Chapel Hill, NC

^3^Department of Pathology, School of Medicine, National and Kapodistrian University of Athens, Athens, Greece

^4^Department of Tumor Microenvironment and Metastasis, H. Lee Moffitt Cancer Center & Research Institute, 12902 Magnolia Drive, Tampa, FL, USA

^5^Department of Pathology and Laboratory Medicine, School of Medicine, The University of North Carolina at Chapel Hill, Chapel Hill, NC, USA

^6^Department of Epidemiology, Gillings School of Global Public Health, The University of North Carolina at Chapel Hill, Chapel Hill, NC, USA

^7^Division of Pediatric and Public Health, Adams School of Dentistry, University of North Carolina at Chapel Hill, Chapel Hill, NC, USA

^8^Division of Hematology/Oncology, University of North Carolina School of Medicine, Chapel Hill, NC, USA

^9^Department of Head and Neck-Endocrine Oncology, H. Lee Moffitt Cancer Center & Research Institute, 12902 Magnolia Drive, Tampa, FL, USA

^10^Department of Pathology, H. Lee Moffitt Cancer Center & Research Institute, 12902 Magnolia Drive, Tampa, FL, USA

^11^Department of Thoracic/Head and Neck Medical Oncology, The University of Texas MD Anderson Cancer Center, Houston, TX, USA

**^*^Corresponding Author:**

Antonio L. Amelio, PhD

Vice Chair for Research in Head and Neck Oncology

Associate Professor and Member

Departments of Tumor Microenvironment and Metastasis and Head and Neck-Endocrine Oncology

H. Lee Moffitt Cancer Center & Research Institute

12902 Magnolia Drive, Tampa, FL 33612

Tel: (813) 745-0905

E-mail: [Antonio.Amelio@moffitt.org](mailto:Antonio.Amelio@moffitt.org)

**Supplemental Information Includes**:

Table S1

Figures S1-S3

**Supplemental Table 1**. List of oligonucleotide primers used for the quantitative RT-PCR assay.

| **Target gene** | **Forward primer (5’-3’)** | **Reverse primer (5’-3’)** | **Amplicon length** |
| --- | --- | --- | --- |
| *NRF2* | ACATCCAGTCAGAAACCAGTG | GGAATGTCTGCGCCAAAAGC | 111 bp |
| *NQO1* | GTGATATTCCAGTTCCCCCT | GGCAGCGTAAGTGTAAGCAAA | 102 bp |
| *TXNRD1* | GCATCCCTGGTGACAAAGAA | GCACTCCAAAGCGACATAG | 110 bp |
| *HMOX1* | AAAGATTGCCCAGAAAGCCCT | ATGCGGGAGCGGTAGAGC | 114 bp |

**
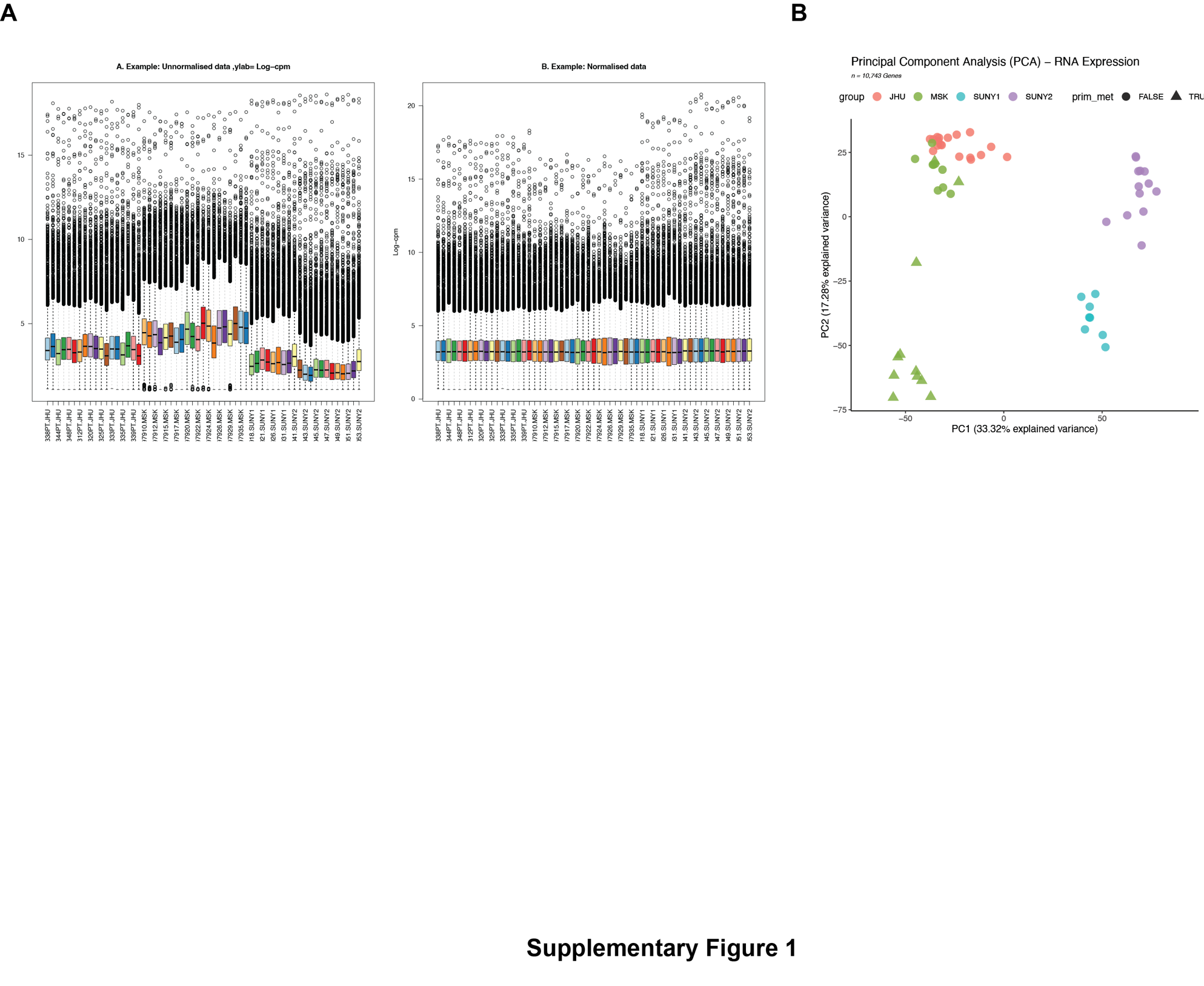
**

**Supplemental Figure S1**. **A**) Bulk RNA sequencing dataset before (*left*) and after (*right*) harmonization. **B**) Direct visualization of harmonized datasets in a principal component analysis (PCA) dimensional reduction plot.

**
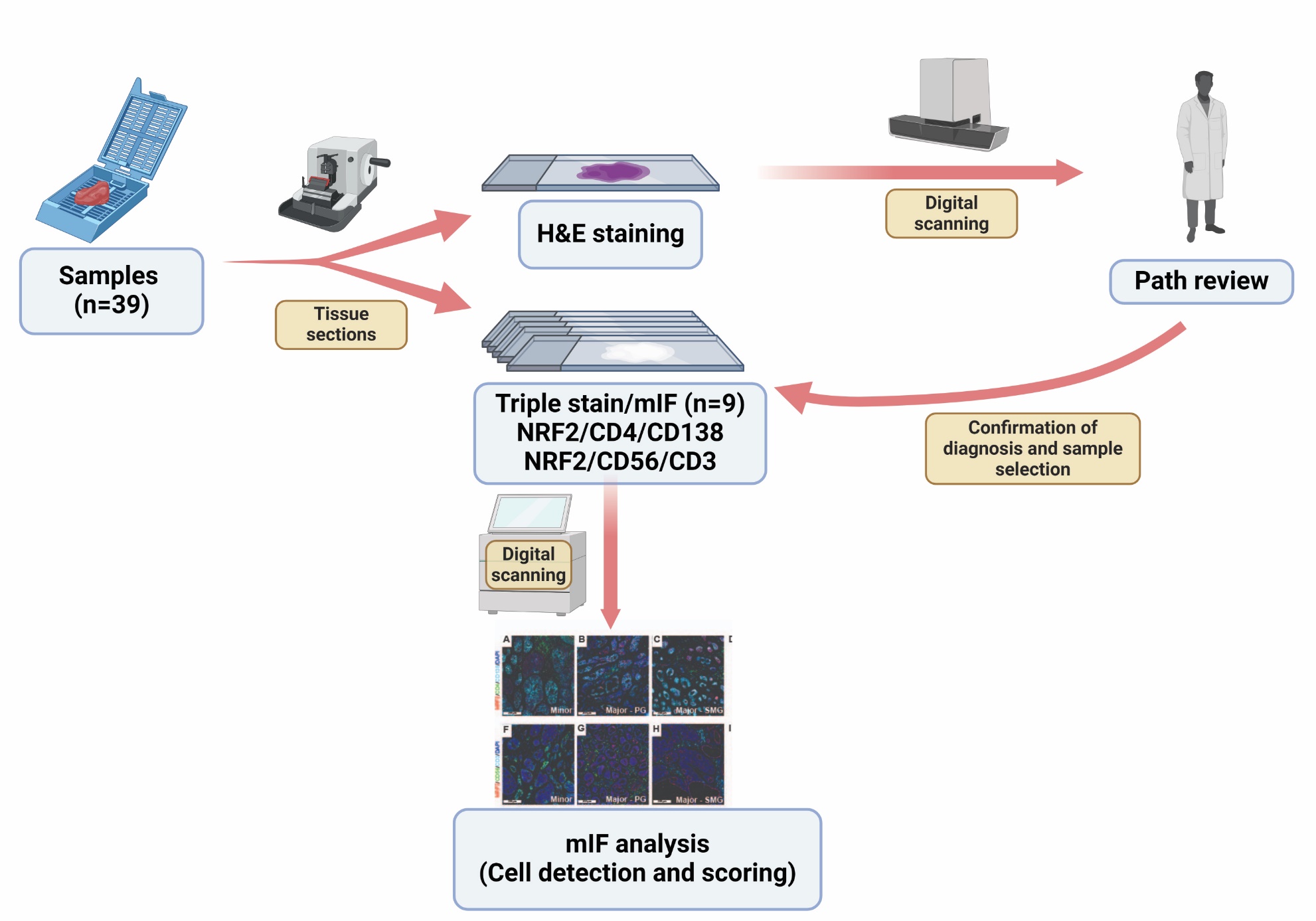
**

**Supplemental Figure S2**. Flowchart for sample identification and multiplex immunofluorescence staining. mIF: multiplex immunofluorescence staining. Figure generated with Biorender.


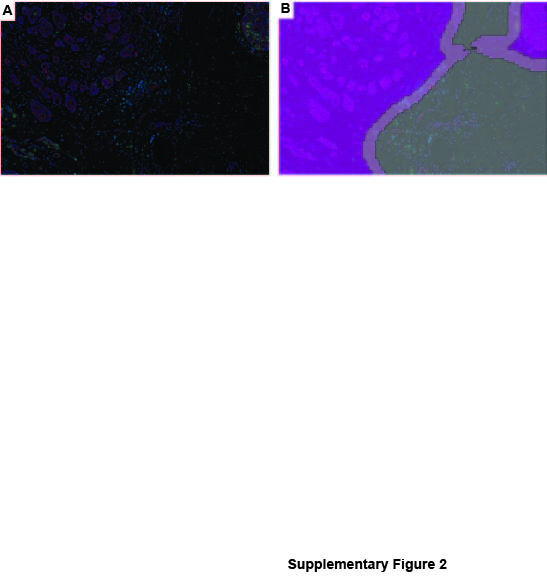


**Supplemental Figure S3**. **A**) Example of manually annotated tumor regions of interest (ROIs). **B**) Algorithm-generated margins surrounding tumor ROIs.
